# Effectiveness of high efficiency particulate (HEPA) air condition combined with the antifungal prophylaxis on incidence, morbidity and mortality of invasive fungal infections in patients with acute myeloid leukemia: a retrospective single-center study

**DOI:** 10.3389/fonc.2024.1429221

**Published:** 2024-10-17

**Authors:** Linda Preyer, Eik Vettorazzi, Walter Fiedler, Holger Rohde, Jannik Stemler, Saskia Gönner, Carsten Bokemeyer, Cyrus Khandanpour, Friederike Wortmann, Maxim Kebenko

**Affiliations:** ^1^ Hubertus Wald Tumorzentrum, Department of Oncology–Hematology, Bone Marrow Transplantation and Pneumology, University Cancer Center, Hamburg, Germany; ^2^ Department for Trauma Surgery, Orthopedics and Hand Surgery Städtisches Klinikum, Solingen, Germany; ^3^ Center of Experimental Medicine, Institute for Medical Biometry and Epidemiology, University Medical Center Hamburg-Eppendorf, Hamburg, Germany; ^4^ Center for Diagnostics, Institute of Medical Microbiology Virology and Hygiene, University Medical Center Hamburg-Eppendorf, Hamburg, Germany; ^5^ Institute of Translational Research, Cologne Excellence Cluster on Cellular Stress Responses in Aging-Associated Diseases (CECAD), University of Cologne, Cologne, Germany; ^6^ Faculty of Medicine, University of Cologne, Cologne, Germany; ^7^ Department I of Internal Medicine, Center for Integrated Oncology Aachen Bonn Cologne Duesseldorf (CIO ABCD) and Excellence Center for Medical Mycology (ECMM), University Hospital Cologne, Cologne, Germany; ^8^ Partner Site Bonn-Cologne Department, German Centre for Infection Research (DZIF), Cologne, Germany; ^9^ Clinic for Hematology and Oncology, University Hospital Schleswig-Holstein, Lübeck, Germany

**Keywords:** HEPA, IFI/EORTC, posaconazole, voriconazole, antifungal drug prophylaxis

## Abstract

**Introduction:**

Our monocentric and retrospective study aimed to investigate the clinical effectivity of HEPA filters in combination with the antifungal drug prophylaxis in patients with AML undergoing intensive chemotherapy and allogeneic stem cell transplantation (SCT).

**Methods/Results:**

We included 177 patients between 2005 and 2015 representing a total of 372 in-hospital stays, 179 in the HEPA cohort (+HEPA) and 193 in the cohort without HEPA filters (-HEPA). No significant additional benefit of HEPA filtration on the risk reduction of IFI was observed. HEPA filtration did not significantly affect the risk of intensive care unit (ICU) admissions or early mortality rates. In patients who received allogeneic SCT in first complete remission with antifungal drug prophylaxis during prior induction treatment, a numerical but not significant improvement in long-term overall survival was noted in the +HEPA cohort compared to the -HEPA cohort (55% to 66%, p = 0.396). For better depicting of the clinical reality, we determined the so-called clinical suspected IFI (csIFI) -defined as cases with antifungal treatment after recommended prophylaxis without fulfilling current EORTC criteria. Especially in patients with a high risk for second IFI, significant risk reduction of csIFI and frequency of ICU admissions was observed when voriconazole was used as secondary antifungal prophylaxis. (csIFI, adjusted effect: OR 0.41, 95% CI (0.21 – 0.82), p = 0.01; csIFI, subgroup-specific effect: OR 0.35, 95% CI (0.15 – 0.78), p = 0.01; ICU, adjusted effect: OR 0.44, 95 CI (0.19 - 1.01), p = 0.05; respectively).

**Discussion:**

In summary, the study suggests the efficacy of secondary antifungal prophylaxis in preventing IFI in AML patients undergoing intensive treatment. The addition of HEPA filtration also demonstrated additional numerous benefits in reducing the frequency of IFI-associated complications.

## Introduction

Acute myeloid leukemia (AML) is a hematological malignancy which is treated with intensive chemotherapy and allogeneic stem cell transplantation (SCT) dependent on the European Leukemia Network (ELN) classification (1). Especially, during intensive remission-induction chemotherapy, treatment-associated prolonged neutropenia predisposes patients to develop IFI with a high morbidity and mortality (2, 3).

The incidence and mortality of invasive fungal infections (IFIs) have decreased in recent years but still pose a significant clinical challenge (4–6).. Aspergillus spp. and Candida spp. remain the most common pathogens in this context (7–9). However, there has been a notable shift in incidence, with Aspergillus spp. becoming more prevalent than Candida spp. over the last few decades (8–11) associated with a mortality of up to 42% (8, 10, 12, 13). Before the widespread use of prophylactic posaconazole, Candida spp. accounted for the majority of IFI cases, often presenting with manifestations such as hepatosplenic candidiasis, fungal endophthalmitis, or acute disseminated candidiasis (14). However, with the introduction of posaconazole, there has been a gradual transition towards pulmonary and ZNS infections caused by Aspergillus spp (5, 6, 13). While most IFIs are not diagnosed *in vivo* (6, 13), post-mortem autopsy studies show that in 24-30% of patients with acute leukemia fungal pathogens can be detected (5, 12). This highlights the high clinical relevance of IFIs and underscores the importance of early and aggressive diagnosis and treatment to improve patient survival (9, 15).

The criteria for categorizing IFIs into possible, probable, or proven cases are defined by the European Organization for Research and Treatment of Cancer/Mycoses Study Group (EORTC/MSG). They base on a combination of factors, including host characteristics, clinical manifestations, microbiological testing, and additional investigations (16, 17). It has been shown that even if the current definitions of the EORTC/MSG are not met, the constellation of host factors (fever, days of neutropenia, coughing etc.) and, especially, detection of multiple CT-lesions are highly associated with IFIs (18–21). A combined approach of different diagnostic tools, such as CT-imaging, PCR- and b-Galactomannan testing, can even lead to a negative predictive value of up to 95%, as stated by various authors reducing the use of antifungal drugs from 29% to 17% (22–24).

In 2007, posaconazole was the first drug to be recommended and approved for use in patients with AML following intensive induction chemotherapy. Its use was subject of many studies in the past. Most of them showed a significant clinical benefit (25–29), especially compared to previous antifungals such as itra- or fluconazole (26). It decreased the incidence of IFI and significantly reduced the absolute mortality at day 100 by 7% prolonging overall survival with a low number needed to treat (26, 28, 30).

Still, problems of azole prophylaxis e.g. drug interactions, toxicity, formation of resistance and the increase of treatment costs remain (31, 32). In view of the numerous organ toxicities, it is currently recommended to determine the serum level of posaconazole (33). Regarding recommended determination of circulating b-Galactomannan, it must be noted that the informative value of this assay can be reduced by simultaneous use of posaconazole (34).

The impact of fungal spores in hospital environments on the incidence of IFI is a matter of ongoing debate (9, 35). To address this issue, many hospitals have implemented HEPA filters to reduce airborne spore concentrations, particularly in wards treating immunocompromised patients such as those with AML. However, the effectiveness of HEPA filtration in reducing IFI incidence, morbidity, and mortality remains controversial, as does the necessity of prophylactic antifungal drug administration alongside HEPA filtration (36–38).

This retrospective, monocentric study was performed in a 1700-bed level 3 university hospital to analyze and to compare the use of HEPA filtration and antifungal drugs on the prevention of IFI. We investigated the prevalence, morbidity and mortality of IFI in AML patients undergoing intensive treatment.

## Methods

We conducted a monocentric retrospective study covering the period between March 2005 and June, 2015 at the University Medical Center Hamburg - Eppendorf (UKE). The inclusion of study participants based on the positive vote of the local ethics committee in accordance with the Hamburger Hospital Law (file number PV7335).

Adult patients with newly diagnosed or relapsed AML who received an intensive induction or consolidation chemotherapy were included in the study. Patients with clinical signs of active pneumonia before treatment initiation as well as patients who were not suitable for intensive therapy were excluded from analysis. If induction therapy led to remission, up to four cycles of consolidation therapy or allogeneic SCT followed.

We explored two different study cohorts. The first main cohort included patients between 2005 and 2011 treated on the leukemia ward without HEPA air condition. Initially, it was divided into the period 2005-2008 without an antifungal prophylaxis and into the period since the approval of posaconazole in 2007/2008 until 2011. However, due to a very small number (n=15) and to a limited evaluability of the archive files, we excluded patients from 2005-2008 from all further analysis. The second main cohort included patients between 2011 and 2015 who received the recommended antimycotic prophylaxis but were treated on the leukemia ward with HEPA air condition.

**Figure 1:**
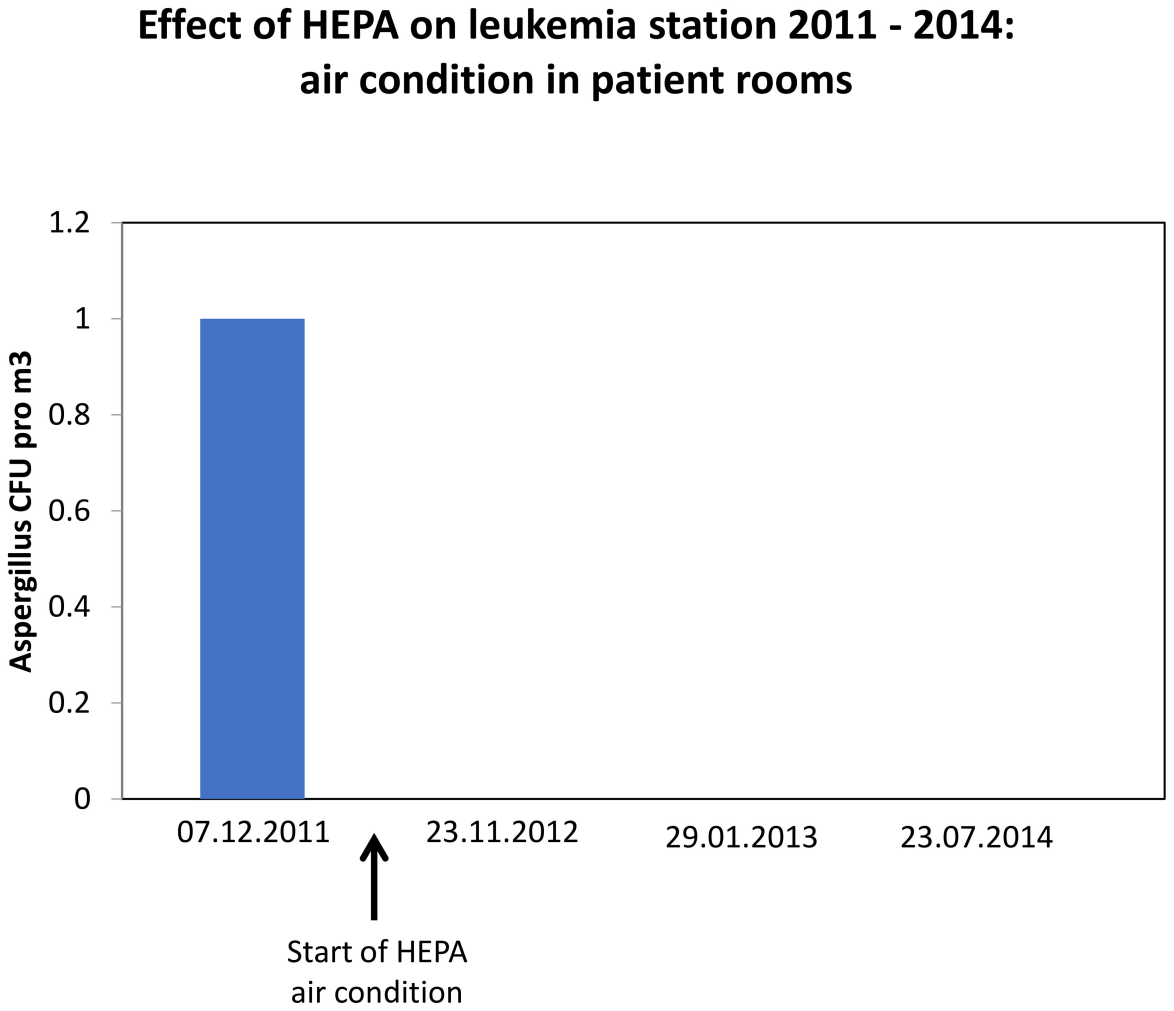
Reduction of fungal spore concentration in air by HEPA condition on leukemia station.

Additionally, we explored the impact of HEPA on IFI-associated mortality in a cohort of patients 2008 – 2015 with newly diagnosed AML and allogeneic SCT in first remission who received primary antifungal drug prophylaxis during induction therapy (**Figure 1**). Selection of this patient cohort based on the fact that patients with a post-remission allogeneic SCT are at a high risk of IFI due to the mostly myeloablative consolidation therapy compared with conventional consolidation therapy (51).

Primary prophylaxis was considered as administration of mainly posaconazole. Secondary prophylaxis included mainly voriconazole and was prescribed after an evident or assumed IFI in order to prevent renewed infections.

IFI events were defined according to the MSG/EORTC 2020 revised criteria as “proven”, “probable” and “possible”, respectively (17, 39). Since the controversial discussion regarding the application of IFI EORTC criteria in clinical practice continues (15), we additionally defined the so called csIFI. This definition included all cases of persistent febrile neutropenia despite broad-spectrum antibiotics leading to an escalation of antifungal therapy which was based on the assessments of the clinicians and did not fully meet EORTC criteria. CT-infiltrates were defined as all pulmonary pathologies suggestive of a fungal infection, such as halo- and air crescent sign as well as caverns.

Sepsis was defined based on the criteria of the “The Third International Consensus Definitions for Sepsis and Septic Shock” (40). Events of sepsis and ICU admissions were defined as all documented cases after a diagnosis of IFI according to EORTC within first 100 days after the start of induction or consolidation treatment.

Based on the recommendations of the Infectious Disease Working Party of the German Society for Hematology and Oncology, we examined the influence of some additional diagnoses, that were under active specific treatment, on the development of IFI: COPD, pulmonary fibrosis, pulmonary arterial hypertension, adipositas, diabetes mellitus, as well as active smoking (41, 42). Further, the influence of blast persistence during induction therapy on the development of IFI was explored.

### Statistics

Patient characteristics were reported as per individual and per hospital admission as counts and percentages for all discrete data, mean and standard deviation for continuous data and were compared between groups using Fisher’s exact test or Mann-Whitney U test as applicable.

Our outcomes were prevalence of IFI, morbidity (sepsis, ICU), early (<100 days starting on the day of hospital admission) and late (5-year overall survival) IFI-associated mortality.

Each patient could have several therapy cycles, which induces a cluster structure with dependent data. We therefore used mixed logistic regression models with patients as random clusters in the analyses of dichotomous outcomes. We used interaction effects in the mixed models to test whether the introduction of HEPA filters had an impact on the effectiveness of the primary or the secondary fungal prophylaxis. If we found no evidence for the presence of an interaction, we simplified the models and reported only the main effects. Moreover, since the HEPA filters were introduced at a specific point in time, all comparisons between these groups are also comparisons to historical data. Thus, basic patient characteristics may also have changed over time. We accounted for this by performing additional analyses in which we adjusted for the relevant characteristics.

For the survival outcome, we analyzed all patients within 100 days as well as for the long-term survival. We used the method of Kaplan-Meier to estimate survival curves and we compared the HEPA groups using a log-rank test. Effects were reported as hazard ratio (HR) and 95% CI from a Cox proportional hazards model.

All analyses were performed using R version 4.2.3. P-values below 0.05 were considered statistically significant. As this was an exploratory study, we refrained from adjusting p-values for multiple testing.

### Results

Between early 2008 and late 2014, data of 169 adult patients with the diagnosis of new or relapsed AML were analyzed in this study. Since 2008, we started applying the primary antifungal prophylaxis with posaconazole according to the international recommendations. In 2011, HEPA air filtration system was put into operation on our leukemia ward.

Overall, 77 patients in the “-HEPA” and 92 in the “+HEPA” cohort were documented (**Table 1**).

**Table 1:** Baseline characteristics of our study population.

Cohort	no HEPA (N=85)	with HEPA (N=92)	total (N=177)	p value
**Age**				0.243^1^
Mean (SD)	55.4 (12.6)	57.2 (13.6)	56.3 (13.1)	
Median (Range)	58.0 (17.0, 74.0)	59.5 (20.0, 78.0)	59.0 (17.0, 78.0)	
**Sex**				0.294^2^
male	40 (47.1%)	51 (55.4%)	91 (51.4%)	
female	45 (52.9%)	41 (44.6%)	86 (48.6%)	
**Year of hopsitalization**				< 0.001^3^
N-Miss	0	1	1	
2005	2 (2.4%)	0 (0.0%)	2 (1.1%)	
2006	1 (1.2%)	0 (0.0%)	1 (0.6%)	
2007	5 (5.9%)	0 (0.0%)	5 (2.8%)	
2008	25 (29.4%)	0 (0.0%)	25 (14.2%)	
2009	7 (8.2%)	0 (0.0%)	7 (4.0%)	
2010	26 (30.6%)	0 (0.0%)	26 (14.8%)	
2011	18 (21.2%)	3 (3.3%)	21 (11.9%)	
2012	1 (1.2%)	24 (26.4%)	25 (14.2%)	
2013	0 (0.0%)	34 (37.4%)	34 (19.3%)	
2014	0 (0.0%)	30 (33.0%)	30 (17.0%)	
**Allogeneic SCT**				0.009^2^
no	52 (61.2%)	35 (38.0%)	87 (49.2%)	
<= d 100	16 (18.8%)	27 (29.3%)	43 (24.3%)	
> d 100	17 (20.0%)	30 (32.6%)	47 (26.6%)	
**Pulmonary co-morbidities**				0.781^2^
N-Miss	22	0	22	
no	58 (92.1%)	83 (90.2%)	141 (91.0%)	
yes	5 (7.9%)	9 (9.8%)	14 (9.0%)	
**Adipositas**				0.578^2^
N-Miss	11	0	11	
no	59 (79.7%)	69 (75.0%)	128 (77.1%)	
yes	15 (20.3%)	23 (25.0%)	38 (22.9%)	
**Diabetes mellitus**				0.795^2^
N-Miss	21	0	21	
no	58 (90.6%)	81 (88.0%)	139 (89.1%)	
jes	6 (9.4%)	11 (12.0%)	17 (10.9%)	
**Fumatorium**				0.132^2^
N-Miss	19	0	19	
no	38 (57.6%)	64 (69.6%)	102 (64.6%)	
yes	28 (42.4%)	28 (30.4%)	56 (35.4%)	

(^1^Kruskal-Wallis rank sum test, ^2^Fisher’s Exact Test for Count Data, ^3^Trend test for ordinal variables).

The cohorts did not significantly differ in terms of age (mean age 55.4 vs 57.2) and sex (53% vs 45% female, 47% vs 55% male), respectively. We found 42% and 30% active smokers, 7.9% and 9.8% of patients with COPD and 11% with diabetes mellitus in -HEPA vs. +HEPA cohort. We observed a significant higher proportion of patients received allogeneic SCT in the “+HEPA” cohort (61% +HEPA vs. 39% -HEPA, p = 0.023).

All included patients contributed to a total of 372 in-hospital stays, 193 in the cohort without HEPA filter, 179 in the cohort with HEPA filter, respectively (**Table 2**). The distribution of the newly diagnosed and of the relapsed AML as well as of the antimycotical primary and secondary drug prophylaxis were not significantly different in -HEPA vs. +HEPA cohort: However, we detected a significant increase in csIFI in the +HEPA vs. -HEPA cohort (**Table 2**).

**Table 2:** Baseline characteristics per hospital stay.

Cohort	no HEPA (N=193)	with HEPA (N=179)	total (N=372)	p value
**Year of hospitalization**				< 0.001^1^
N-Miss	0	1	1	
2005	3 (1.8%)	0 (0.0%)	3 (0.9%)	
2006	2 (1.2%)	0 (0.0%)	2 (0.6%)	
2007	6 (3.5%)	0 (0.0%)	6 (1.7%)	
2008	41 (24.1%)	0 (0.0%)	41 (11.8%)	
2009	21 (12.4%)	0 (0.0%)	21 (6.0%)	
2010	48 (28.2%)	0 (0.0%)	48 (13.8%)	
2011	46 (27.1%)	7 (3.9%)	53 (15.2%)	
2012	2 (1.2%)	41 (23.0%)	43 (12.4%)	
2013	0 (0.0%)	61 (34.3%)	61 (17.5%)	
2014	1 (0.6%)	65 (36.5%)	66 (19.0%)	
2015	0 (0.0%)	4 (2.2%)	4 (1.1%)	
**AML diagnosis**				1.000^2^
new	152 (89.4%)	161 (89.9%)	313 (89.7%)	
r/r	18 (10.6%)	18 (10.1%)	36 (10.3%)	
**Drug prophylaxis**				1.000^2^
Primary	115 (67.6%)	121 (67.6%)	236 (67.6%)	
Secondary	55 (32.4%)	58 (32.4%)	113 (32.4%)	
**Blast persistence**				0.554^2^
N-Miss	16	7	23	
no	101 (65.6%)	119 (69.2%)	220 (67.5%)	
yes	53 (34.4%)	53 (30.8%)	106 (32.5%)	
**Escalation of antifungal treatment**				< 0.001^2^
no	123 (72.4%)	89 (49.7%)	212 (60.7%)	
yes	47 (27.6%)	90 (50.3%)	137 (39.3%)	
**ECOG >2**				0.191^2^
N-Miss	29	0	29	
no	120 (85.1%)	141 (78.8%)	261 (81.6%)	
yes	21 (14.9%)	38 (21.2%)	59 (18.4%)	

(^1^Trend test for ordinal variables, ^2^Fisher’s Exact Test for Count Data).

**Figure 2:**
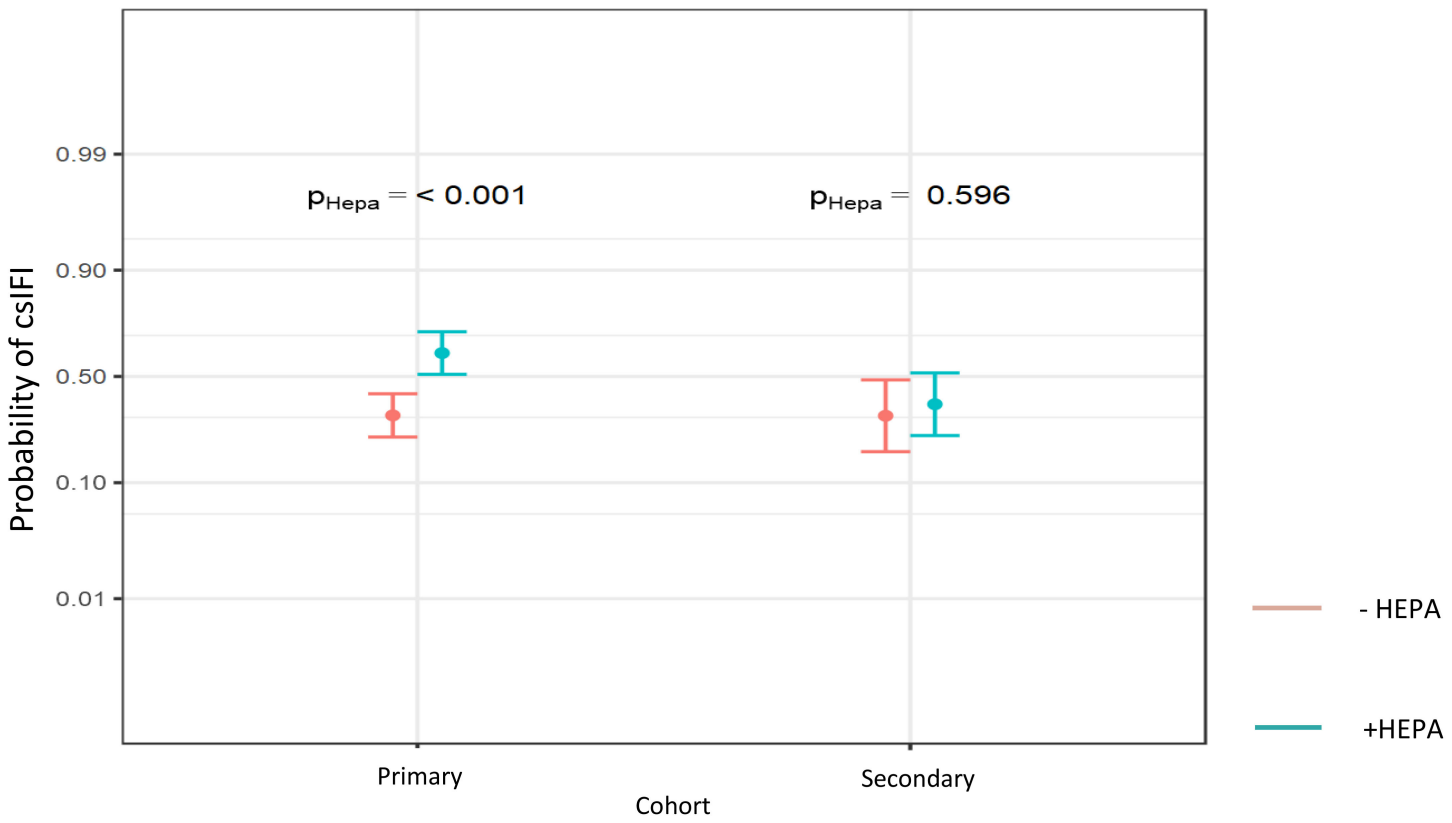
Probability of csIFI in dependence of HEPA within the different antifungal drug prophylaxis cohorts (Probability values are reported, significance level P < 0.05).

Between 2008 and 2015, 108 patients with newly diagnosed AML and allogeneic SCT in first remission were included, compromising 69 in the + HEPA and 39 in the -Hepa cohort respectively. (**Figure 2B**, s. “Supplements”).

No air contamination with fungal spores (measured as colony forming air units (CFU) pro m3) in patient rooms was detected since HEPA filters became operational in December 2011 (**Figure 1**). To detect specific effects of HEPA on IFI, we used statistical interaction models. We found *a significant* increase of csIFI by HEPA in patients with the primary *prophylaxis* compared with those without HEPA. In congruence with these findings, the probability of csIFI was significantly higher in patients with primary prophylaxis using HEPA compared to those without HEPA. In the secondary prophylaxis cohort, this probability did not change significantly (30% vs 61%, OR 3.63, 95% CI (1.92 -6.86), p < 0.001; 30% vs 36%, OR 1.27, 95% CI (0.52 – 3.07), *p = 0.*596, respectively*;* **Tables 3A–C**, **Figure 2**).

**Table 3:** Specific main and interaction effects of HEPA and antifungal drug prophylaxis on csIFI.

A				
Indep. Variable (in all included cases)	Odds Ratio	95% CI	p-value	
Target: csIFI				
+HEPA vs. –HEPA	2.67	1.53 - 4.64	< 0.001	
Secondary vs. primary prophylaxis	0.54	0.28 - 1.03	0.063	
B				
Indep. Variable (in all included cases)	Odds Ratio	95% CI	p-value	
Target: csIFI				
+HEPA vs. -HEPA	1.75	1.00 - 3.05	0.049	
Secondary vs. primary prophylaxis	0.41	0.21 - 0.82	0.012	
Blast persistence	0.9	0.52 - 1.57	0.714	
Pulmonary co-morbidities	0.84	0.37 - 1.94	0.687	
Diabetes mellitus	0.92	0.41 - 2.07	0.838	
Adipositas	0.62	0.32 - 1.18	0.147	
Fumatorium	1.2	0.68 - 2.13	0.524	
C				
Stratum	Contrast	Odds Ratio	95% CI	p-value
Target: csIFI				
Primary prophylaxis	+HEPA vs. -HEPA	3.63	1.92 - 6.86	< 0.001
Secondary prophylaxis	+HEPA vs. -HEPA	1.27	0.52 - 3.07	0.596
Target: csIFI				
-HEPA	Secondary vs. primary prophylaxis	0.99	0.43 - 2.27	0.985
+HEPA	Secondary vs. primary prophylaxis	0.35	0.15 - 0.78	0.011

A: main unadjusted effects: Interaction of HEPA filter with antifungal drug prophylaxis in reducing the risk of csIFI. B: adjusted effects: statistical impact of different variables on the risk of csIFI. C: subgroup-specifc effects of HEPA and of the antifungal drug prophylaxis are reported (significance level, p= 0.048).

The effectiveness of the currently recommended antimycotic drugs for prevention of csIFI in the -/+HEPA cohort was also explored. We were only able to compare the risk of csIFI under secondary with that under primary antifungal prophylaxis - due to the following reasons. First, as mentoined above, we did not include patients any without antifungal drug prophylaxis in our analysis due to the incomplete data files, so no control-cohort data could be collected for comparative analysis of the effectiveness of posaconazole. Secondly, if patients developed csIFI under the primary prophylaxis, the application of the secondary prophylaxis should be ideally expected in all these cases. However, we did not observe any differences in the distribution of the secondary prophylaxis in the -HEPA vs. +HEPA cohort (32% vs. 32%, *p =* 1.0), although the frequency of csIFI varied strongly: 27.6% by -HEPA vs. 50.3% by +HEPA, p < 0.001 (**Table 2**). Due to this discrepance, no statistically meaningful exploration of the risk of csIFI in patients without vs. with the secondary prophylaxis would have been possible. Given that, we found a numerical reduction of the risk of csIFI by the secondary compared to the effect of the primary antifungal prophylaxis in the unadjusted model. However, the significance level was marginally not reached (*OR* 0.54, 95% CI (*0.28 – 1.03*), p = 0.06, **Table 3A**). Using adjusted and interaction model, a significant higher reduction of csIFI by the secondary prophylaxis could also be observed in the overall, and particularly, in the + HEPA cohort (OR 0.41, 95% CI *(*0.21 – 0.82*)*, p = 0.01; **Table 3B** and OR 0.35, 95% CI *(*0.15 – 0.78*)*, p = 0.01; **Table 3C**, respectively).

**Figure 3:**
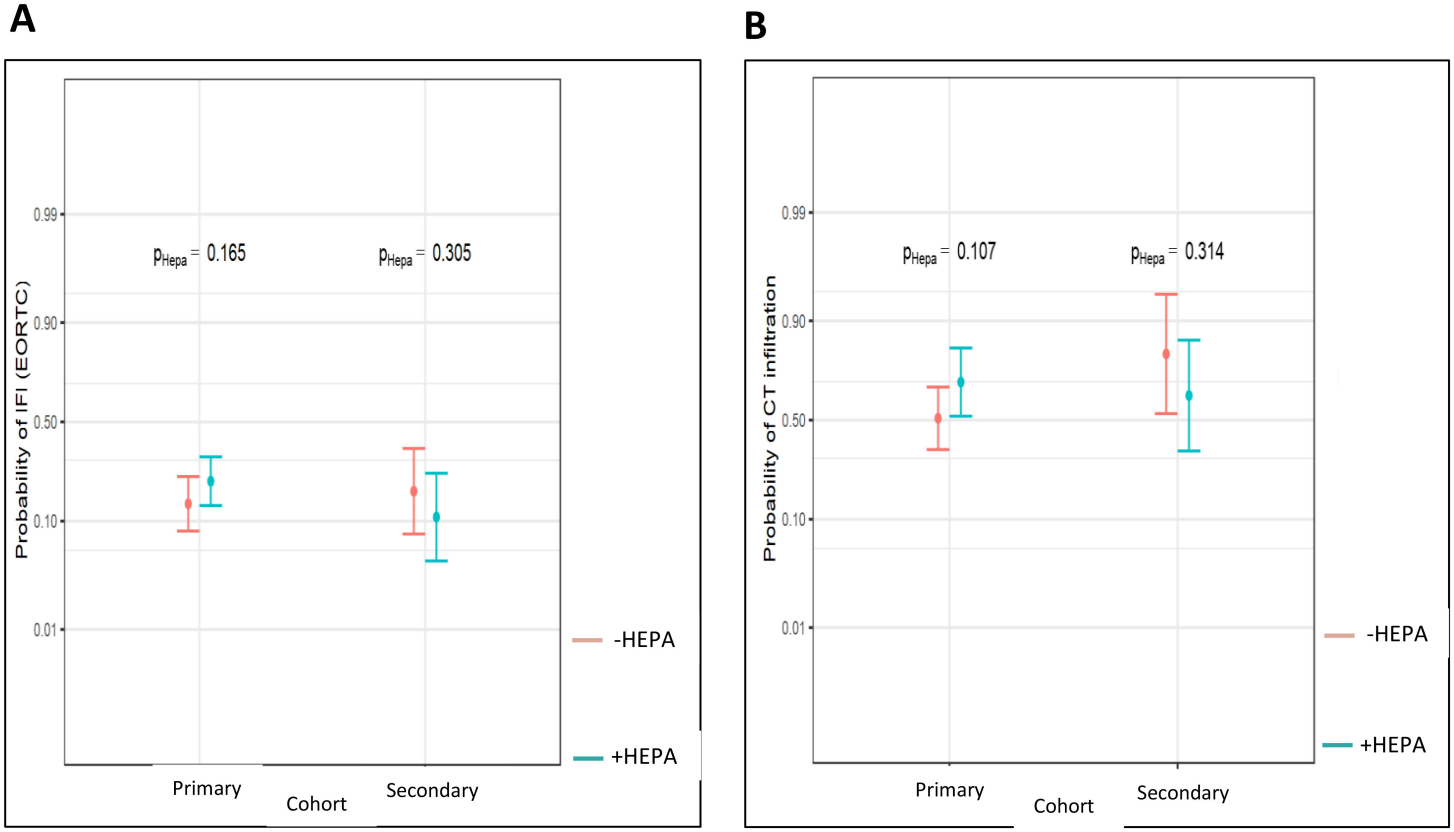
Probability of IFI according to EORTC **(A)** and of pulmonary infiltrates **(B)** within the different antifungal drug prophylaxis cohorts (Probability values are reported, significance level P < 0.05).

To account the IFI definition according to the current EORTC/MSG criteria, which are commonly used in the clinical studies, we also analyzed the same statistical parameters regarding the risk of IFI according to EORTC. When applying strict EORTC criteria, *we* were not able to find any significant effects neither of HEPA nor of the antifungal prophylaxis on IFI in our retrospective and not-randomzied data set (**Table 4**, **Figure 3A**). Secondly, the probability of pulmonary infiltrates, as a separate IFI indicator, was also not significantly reduced by HEPA or by the antimycotic prophylaxis (**Table 4**, **Figure 3B**).

**Table 4:** Specific effects of HEPA, antifungal drug prophylaxis and other clinical variables on the IFI according to EORTC and development of CT-infiltrats.

A			
Indep. Variable (in all included cases)	Odds Ratio	95% CI	p-value
**Target: IFI according to EORTC**			
+HEPA vs. -HEPA	1.21	0.65 - 2.26	0.544
Secondary vs. primary prophylaxis	0.73	0.36 - 1.47	0.376
**Target: Pulmonary infiltrats**			
+HEPA vs. -HEPA	1.53	0.65 - 3.63	0.33
Secondary vs. primary prophylaxis	1.81	0.68 - 4.80	0.236
B			
Indep. Variable (in all included cases)	Odds Ratio	95% CI	p-value
**Target: IFI_according to EORTC**			
+HEPA vs. –HEPA	0.89	0.50 - 1.58	0.691
Secondary vs. primary prophylaxis	0.62	0.31 - 1.23	0.172
Blast persistence	0.81	0.43 - 1.54	0.52
Pulmonary co-morbidities	1.36	0.56 - 3.26	0.496
Diabetes mellitus	0.69	0.28 - 1.73	0.431
Adipositas	0.82	0.42 - 1.63	0.578
Fumatorium	0.66	0.35 - 1.22	0.184
**Target: Pulmonary infiltrats**			
+HEPA vs. –HEPA	1.09	0.50 - 2.40	0.825
Secondary vs. primary prophylaxis	1.05	0.45 - 2.50	0.903
Blast persistence	1.37	0.59 - 3.15	0.463
Pulmonary co-morbidities	1.27	0.45 - 3.59	0.655
Diabetes mellitus	0.61	0.20 - 1.81	0.369
Adipositas	0.51	0.19 - 1.37	0.183
Fumatorium	1.04	0.46 - 2.38	0.923

A: main unadjusted effects, significance level P < 0.05. B: adjusted effects, significance level P < 0.05.

**Figure 4:**
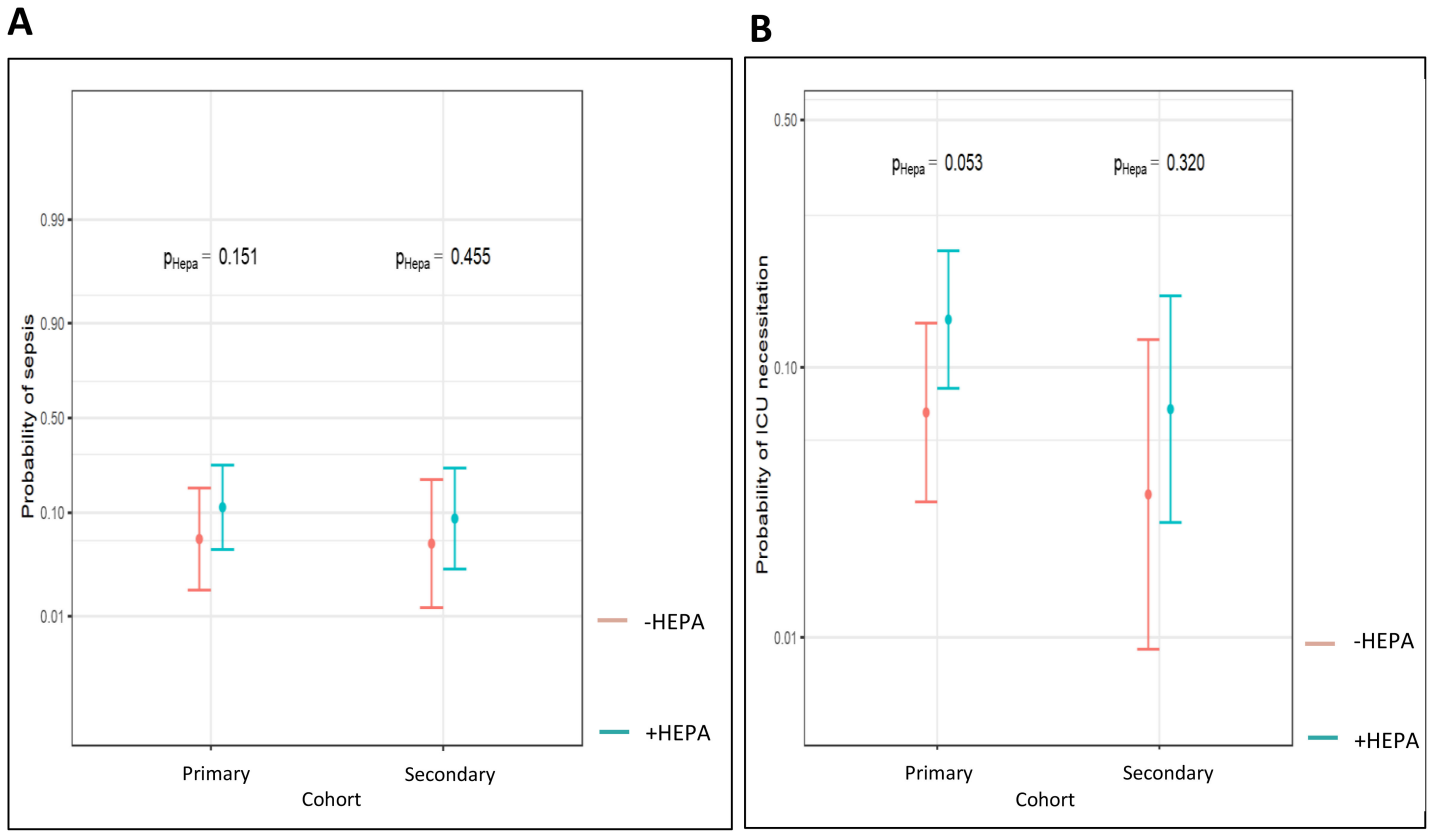
Probability of IFI-associated sepsis **(A)** and ICU **(B)** in dependence of HEPA within the different antifungal drug prophylaxis cohorts (Probability values are reported, significance level P < 0.05).

Further, we investigated a possible link between HEPA, the antifungal drug prophylaxis and the risk of bacterial sepsis as well as the probability of ICU admission - in terms of the secondary, IFI-triggered events. No significant interactions between these parameters could be observed (**Table 5**). The probability of sepsis was not significantly changed by HEPA neither in the primary nor in the secondary prophylaxis cohort (5,7% vs. 11,2%, OR 2.09, 95% CI (0.76, 5.70), p = 0.151; 5.2 vs. 8.9%, OR 1.79, 95% CI (0.39, 8.20), p = 0.455, respectively; **Figure 4A**).

**Table 5:** Specific effects of HEPA, antifungal drug prophylaxis and other clinical variables on IFI-associated sepsis and ICU.

A			
Indep. Variable (in all included cases)	Odds Ratio	95% CI	p-value
Target: Sepsis			
+HEPA vs. -HEPA	2	0.83 - 4.81	0.122
Secondary vs. primary prophylaxis	0.81	0.35 - 1.86	0.625
Target: ICU			
+HEPA vs. -HEPA	2.47	0.20 - 30.32	0.479
Secondary vs. primary prophylaxis	1.38	0.22 - 8.50	0.731
B			
	Odds Ratio	95% CI	p-value
Target: Sepsis			
	Odds Ratio	95% CI	p-value
+HEPA vs. –HEPA	1.4	0.68 - 2.87	0.363
Secondary vs. primary prophylaxis	0.75	0.37 - 1.54	0.436
Blast persistence	1.03	0.51 - 2.10	0.936
Pulmonary co-morbidities	1.11	0.40 - 3.08	0.848
Diabetes mellitus	0.47	0.14 - 1.54	0.21
Adipositas	1.12	0.51 - 2.46	0.787
Fumatorium	1.04	0.52 - 2.09	0.916
Target: ICU			
+HEPA vs. –HEPA	1.51	0.71 - 3.20	0.285
Secondary vs. primary prophylaxis	0.44	0.19 - 1.01	0.052
Blast persistence	0.69	0.32 - 1.47	0.339
Pulmonary co-morbidities	1.11	0.39 - 3.16	0.851
Diabetes mellitus	1.01	0.36 - 2.85	0.983
Adipositas	0.91	0.41 - 2.06	0.828
Fumatorium	0.92	0.44 - 1.91	0.823

A: main unadjusted effects, significance level P < 0.05. B: adjusted effects, significance level P < 0.05.

The probability of ICU admission was also not significantly influenced by HEPA in both antifungal prophylaxis cohorts (6,9% vs. 14,5%, OR 2.28, 95 CI (0.99, 5.27), p = 0.053; 3,5% vs. 7,1%, OR 2.13, 95 CI (0.48, 9.49), p = 0.32, respectively; **Figure 4B**). However, we found a strong numerical reduction of the risk of ICU admission in patients with the secondary, compared with the primary prophylaxis, which almost reached the level of the statistical significance (OR 0.44, 95 CI (0.19 - 1.01), p = 0.052; **Table 5**).

**Figure 5:**
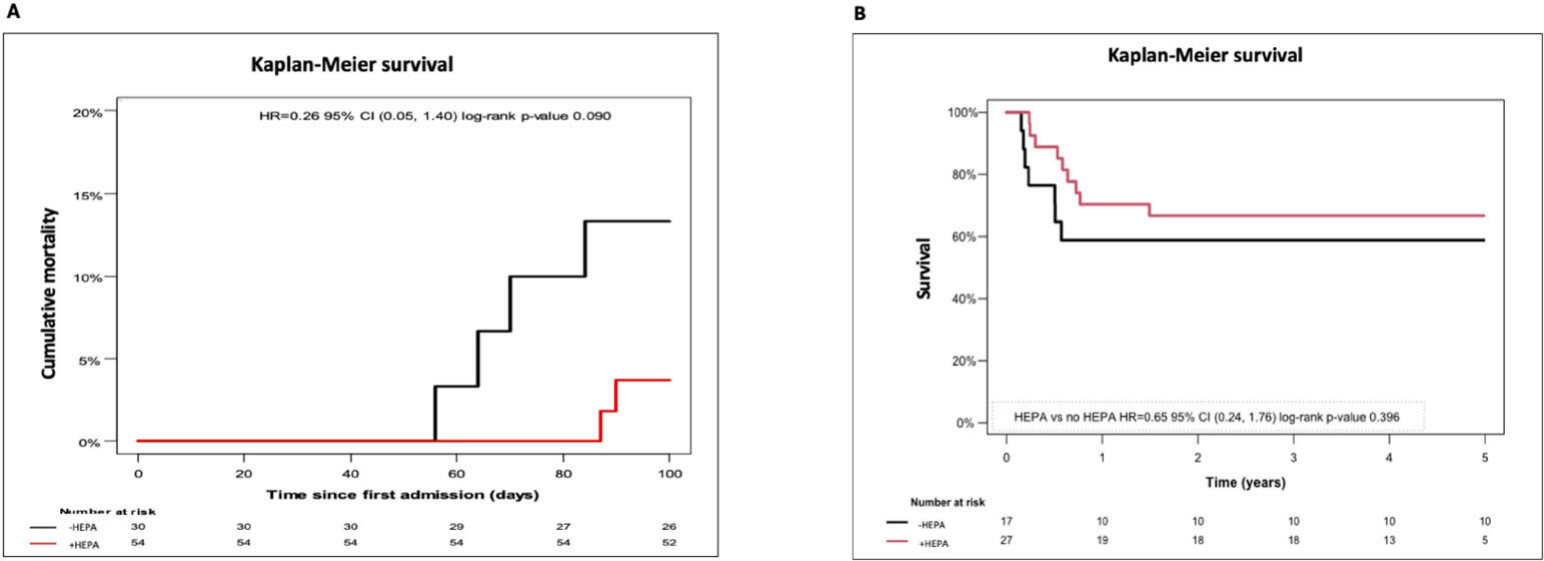
Exploration of the early and the long-term survival in dependence of HEPA filter **(A)** cumulative early mortality in all patients developing IFI defined to EORTC under antifungal drug prophylaxis. **(B)** estimated 5y-overall survival in patients undergoing allogeneic SCT in 1. complete remission (significance level P < 0.05).

The risk of death until day 100 was also numerically reduced from 12% in patients with the antifungal drug prophylaxis alone to 3% in patients with both, the antifungal drug prophylaxis and HEPA. However, the difference was not significant (OR 0.26; p=0.09; **Figure 5A**). In addition, the increase of mortality in the - HEPA cohort notably occurred earlier in average than in the +HEPA cohort: between day 50-60 vs. day 80-90 after intensive pretreatment. No statistically significant impacts of HEPA filter as well as of the antifungal drug prophylaxis on the day 100 mortality could also be shown when the unadjusted model was used (Table 6, s. “Supplements”). In the cohort of patients with newly diagnosed AML and with the *a priori* indicated allogeneic SCT in first complete remission, we likewise did not find a significant additional benefit of HEPA filter on the long-term overall survival after allogenic SCT: it was 55% in the +HEPA vs. 66% in the -HEPA cohort (p = 0.396, **Figure 5B**).

We noted no statistically significant impact of clinical co-morbidities mentioned above (s. “Methods”) on the risk of IFI according to EORTC. Although it is known that blast persistence which leads to prolonged neutropenia as well as allogeneic SCT with inherent risk of GvHD can promote IFI, we were not able to detect significant effect on the risk of IFI when used EORTC criteria. Comparably, we were not able to detect any significant effects of these factors on csIFI, pulmonary infiltrates, ICU, sepsis or on the early IFI-associated mortality. (**Tables 3B**, **4B**, **5B**, respectively).

## Discussion

Optimization of antifungal prophylaxis in AML patients receiving intensive chemotherapy has long been the subject of controversy. In particular, in the age of well-validated recommendations for antifungal drugs, especially for posaconazole, the question arises as to an additional benefit of the high-performance, but also costly, physical processes such as HEPA air condition.

We investigated the role of the HEPA filtration on the prevalence of IFI as well as on the IFI-associated morbidity and mortality in patients with AML who underwent intensive treatment compared to a similar cohort with the azole prophylaxis alone.

In 2003, an examination of fungal spores in the air and of the incidence of IFI during a period of construction on our stem cell transplantation ward equipped with HEPA air condition was done (43). The IFI incidence was historically compared to that of patients treated outside construction activity. A strong reduction of fungal spore concentration inside the ward (incl. patient rooms) translated into a reduction of IFI in allogeneic stem cell recipients using HEPA filters could be shown. On the other hand, Krüger et al. noticed a rapid decline in fungal spores outside and inside the transplantation station immediately after the completion of the construction work (44, 45). However, no comparison of the IFI incidence with vs. without HEPA filters outside construction was made in this context, which is why the question of an additional clinical benefit of HEPA filters outside of construction activity remains.

Our examinations also confirm a positive effect of HEPA air condition on the leukemia ward (**Figure 1**): we were not able to detect any fungal spores in patient rooms after HEPA filters had been installed. Contrary to the positive results of some groups, which mainly explored patients who did not receive prophylaxis with antimycotics before the approval of posaconazole in 2007 (36), we were not able to show a statistically significant benefit in preventing IFI and IFI-related complications by HEPA, when patients concomitantly received antimycotic prophylaxis. In 2023, Dührsen et al. also did not find any difference in mortality between +HEPA and -HEPA cohort when posaconazole prophylaxis was used (46). On the contrary, a earlier systematic review from 16 studies including exploration of HEPA use in patients with azole prophylaxis suggests at least some benefit on the mortality or prevalence of IFI in highly immunosuppressed patients due to environmental control (38). To underline the controversy of this topic, in 2013 Menequeti et al. showed in an integrative review of 13 studies a decrease in CFU values with ensuing decrease in fungal infection and associated mortality, however, primarily in patients undergone allogeneic SCT (37).

One of the hypotheses to explain lacking significant effects of HEPA on the early mortality in our report is, that, although the rate of the new IFIs is expected to be reduced by an extremely low fungal load in patient rooms, the majority of IFIs is probably caused by prior endogenous colonization with fungal spores breaking through under prolonged neutropenia and immunosuppression (42, 43). Nevertheless, the numerical reduction of the cumulative early mortality from 12% to 3% (p=0.09), its 30-day later increase and the numerical improvement of the 5-year overall survival from 55% to 66% in patients after allogeneic SCT in the + HEPA cohort should be mentioned. The high individual prophylactical effectiveness of azoles and of HEPA and the limited number of cases on the other hand may probably be seen as the main reasons why statistical significance was not achieved. Our observations are in line with reported data of long-term survival in young patients with AML (47).

Surprisingly, we observed a strong increase of csIFI in the + HEPA cohort (p< 0.001), that, however, did not correlate with the IFI probability defined to EORTC criteria in this cohort. This observation confirms again the problematic nature of the current EORCT criteria in identifying patients with the real need of antifungal treatment making necessary an accurate and critical discussion of therapy indications. Regarding examination of our data, it should be discussed as an “over treatment” bias in the + HEPA cohort, which could limit the interpretation of clinical HEPA effects.

We noted a remarkable decrease of the historically incidence of IFI according to EORTC criteria of ca. 30%, when azole prophylaxis was not yet used (4), to 12% in our posaconazole cohort (**Figure 3A**). Due to the lack of a control cohort, no statistically relevant explorations of the effectiveness of posaconazole in preventing IFI could be done in our study.

Secondary prophylaxis is widely used, based on the fact of a 3-hold higher rate of renewed IFI in patients with pre-treated AML (48). In our study, a significant reduction of application of systemic antifungals in patients, who were prophylactically treated with voriconazole, could be detected (**Tables 3B, C**). In congruence, we found a comparable probability of csIFI of 30% in patients with the secondary prophylaxis, which was without any increase compared with the primary prophylaxis cohort. For patients in the + HEPA cohort, we even observed a strong reduction of csIFI from 61% to 36% by voriconazole (**Figure 2**), which should be interpreted cautiously - given a possible overtreatment bias. The use of the secondary prophylaxis also led to a strong reduction of the IFI associated morbidity (number of ICU stays), which is mainly based on a lower frequency of clinically Aspergillus pneumonia (**Table 5B**). However, we did not see any decline in the number of neutropenic sepsis which suggests a multifactorial mechanism of sepsis in immunosuppressed patients (49). We also found no significant impact of the antifungal azole prophylaxis on the IFI related early mortality.

For the risk factors of various co-morbidities, e.g. COPD or diabetes, active smoking and refractory AML, we were not able to find any significant impact neither on the incidence of the IFI nor on the IFI-associated secondary events in our study population. However, due to the small number of evaluable cases and considering numerous publications that show a significantly higher incidence of IFI in the presence of such risk factors, these data should be assessed with caution.

Considering historical data and our results, the physical and medicamentous antifungal prophylaxis is useful in patients with AML undergoing intensive treatment. Especially, in patients who already had IFI during prior treatment of AML, use of voriconazole to prevent second IFI can be suggested. Further investigations in larger patient cohorts are required to statistically demonstrate the effectiveness of HEPA air conditioning for IFI prevention.

### Limitations of this study

The limited evaluability of the archive files of patients with no antifungal prophylaxis (the subcohort 2005-2008) generally does not allowed us to make any comparative analysis between the cohorts “with vs. without” antimycotic prophylaxis.

The unexplained increase of escalation of antifungal treatments by patients in the + HEPA cohort who received posaconazole prophylaxis and did not meet IFI criteria according to EORTC: it should be included in the interpretation of the morbidity and mortality data in the + HEPA cohort as a possible “over treatment” bias.

Another limitation is that the HEPA filters were installed at a fixed time in our clinic. All comparisons between +HEPA and -HEPA are therefore, with a few exceptions from patients at the time of installation, comparisons to a historical cohort, which can therefore also be biased by external circumstances that were not measured or even measurable.

A randomized study would be necessary to overcome this limitation.

## Conclusion

Our study provides statistical evidence for use of secondary antifungal azole prophylaxes to prevent renewed IFI as well as its complications in patients with AML and intensive pre-treatment. Use of HEPA filtration led to a strong reduction of fungal exposure as well as to a numerically decrease of IFI-associated complications on our leukemia ward.

## Data Availability

The original contributions presented in the study are included in the article/supplementary material. Further inquiries can be directed to the corresponding author.
