# Supplementary material for: Effectiveness of high efficiency particulate (HEPA) air condition combined with the antifungal prophylaxis on incidence, morbidity and mortality of invasive fungal infections in patients with acute myeloid leukemia: a retrospective single-center study
Source: Front Oncol. 2024 Oct 17;14:1429221. doi: 10.3389/fonc.2024.1429221 (PMC11524928; doi:10.3389/fonc.2024.1429221)
Supplement: Supplementary file 1 [file DataSheet1.docx]

**Supplementary Figure 1**: population of patients with de novo AML

with vs. without Allo-BMT in the cohorts primary, secondary antifungal prophylaxis, with vs. without HEPA filter, respectively.

**
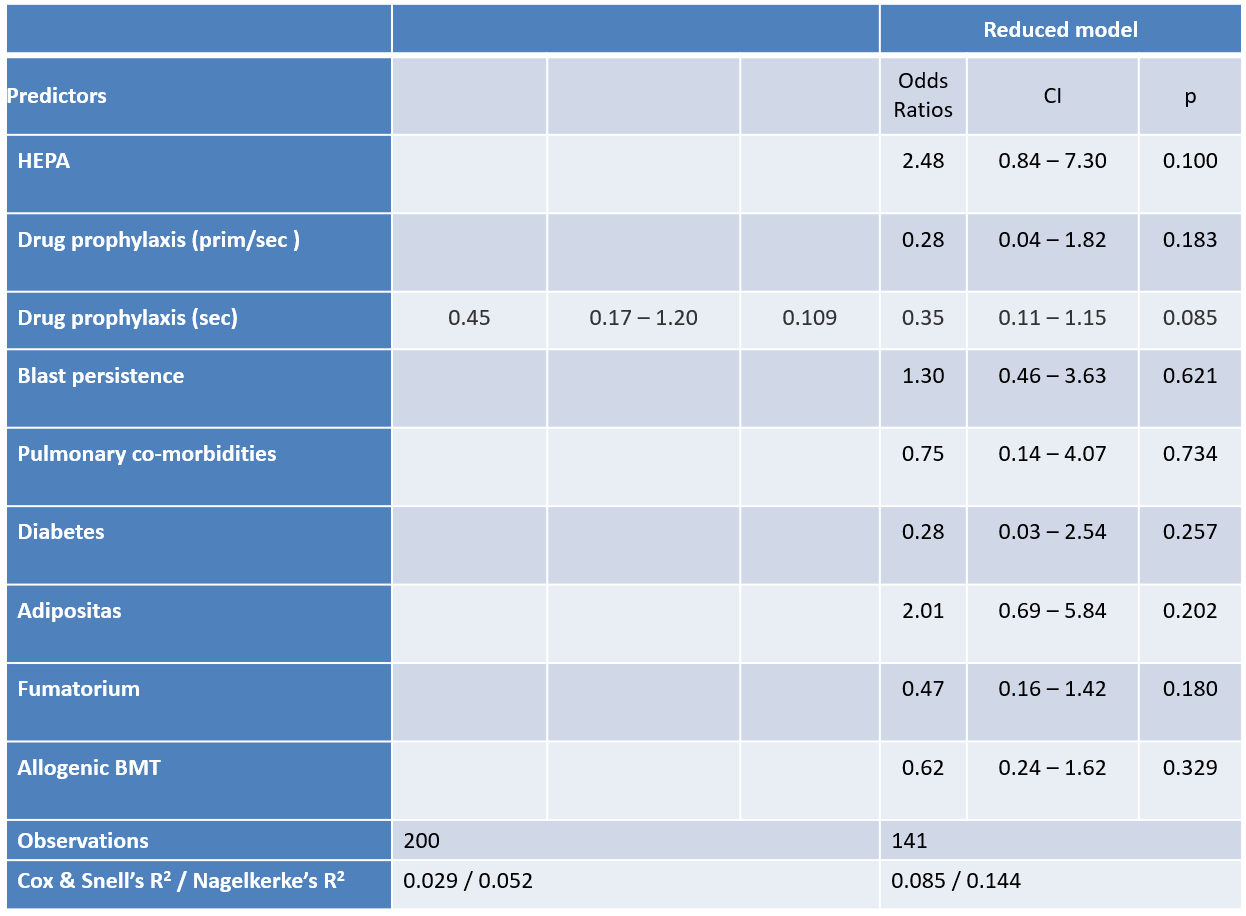
**

**Supplementary Table 1:**

Statistical impact of antifungal drug prophylaxis, HEPA filter and different co-morbidities on the risk of death until day 100
